# Tenotomy as a Preliminary Procedure for the Reduction and Cure of Talipes—Report of Eight Cases*Read at meeting of the Austin District Medical Society, June 26, 1903.

**Published:** 1903-08

**Authors:** J. S. Wooten

**Affiliations:** Austin, Texas


For Texas Medical Journal.

### Tenotomy as a Preliminary Procedure for the Reduction and Cure of Talipes—Report of Eight Cases.*

BY J. S. WOOTEN, M. D., AUSTIN, TEXAS.

The operations for the reduction and cure of talipes may be considered under the following heads:

1. Manual correction, gradual or forcible, with or without an anesthetic.

2. The employment of mechanical force under an anesthetic by means of a tarsoclast, the Thomas wrench, or some form of extension apparatus as used by Schaffer or Phelps.

3. The subcutaneous tenotomy and mynotomy, followed by the employment of manual or mechanical force.

4. The open section, known as the Phelps operation, with the division of all the soft parts until the deformity can be overcome by manual force.

5. Osteotomy, to overcome the more aggravated deformities or those occurring in older cases.

The treatment of club-foot by mechanical force is as old as Hippocrates himself, and successful results have followed the laborious efforts of those early pioneers in orthopedic surgery before the newspaper came to herald their marvelous cures. Some of you, as with the writer, who have interested yourselves in this line of surgery and are familiar with the history of these old operative procedures and their modifications in modern surgery, have been doubtless surprised at the recent commotion and cry that has been raised over the *new* and unheard of bloodless operation for club-foot, and equally chagrined that some of our professional brothers should have mounted the newspaper band wagon and proceeded to ride in the up-to-date procession of public sensationalism and notoriety.

The causation of these congenital deformities is still shrouded in doubt, but the most probable and plausible explanation of their occurrence is the theory of retarded rotation, propounded first by Esserricht and elaborated by Berg, of New York, and Parker, of Lon-

*Read at meeting of the Austin District Medical Society, June 26, 1903.

don. Berg has shown that the position of the feet in the early months of utero-gestation is that of equino varus, and their normal rotation is interfered with by numerous possible causes, and the feet thus failing to unfold are left in the characteristic distortion of varying degrees. The pathology of this deformity is too well known to discuss; the changes involve not alone the ligaments, tendons and muscles, but the bones as well, and it is this last physical and altered condition that I shall specially call your attention to later. Surgical authorities from time immemorial have called our attention to the great good to be accomplished in overcoming the feet deformities in infants by manual treatment applied daily, either by the physician, nurse or mother, and doubtless many of you have seen such cases of mild talipes so corrected. During these early months, before ossification has taken place and while the soft parts are elastic and easily overcome, over-correction in one sitting can be readily attained by manual force and the position of the foot maintained by properly adjusted plaster paris dressing. Such were the procedures practiced in the early days before antiseptic and aseptic surgery. In view of the fact that such cases are usually left alone and neglected and no treatment undertaken, the majority of such deformities usually reach a surgeon at a much more advanced age. In the transactions of the Hospital for Ruptured and Crippled, New York City, 1890, as collected by Dr. W. R. Townsend (in which hospital the writer enjoyed the privilege of working during the summer of 1891), out of two thousand three hundred and eighty-six cases, analysis shows some very interesting facts; that club-foot is among the most frequent congenital deformities; that congenital club-foot is less frequent than non-congenital; that talipes equino varus constitutes three-fourths of all cases; that equinus and calcaneus are rare as congenital deformities, but common as paralytic. It is this larger class of congenital talipes equino varus that I would consider today, and the application of the principles herein considered will be modified according to the experience and judgment of the surgeon in handling the other varieties of talipes.

The method of treatment employed by the writer consists of tenotomy of the tendons of the tibialis anticus and posticus through open incisions made over the tendons at the lower end of the tibia as they course through the anterior and posterior grooves of the internal malleolus, together with subcutaneous tenotomy of the tendo-achilles and forcible massage. The relative position of the first two tendons is first made out and a small incision carried down through the fascia, the tendon raised out of its channel by means of a strabismus hook, the identity of the tendon thoroughly established, and then divided. No difficulty is experienced in hooking up the anterior, but owing to the deeper groove occupied by the tendon of the posticus muscle, trouble is sometimes experiençed, but which may be overcome by having the assistant sharply adduct and rotate the foot in order to relax this tendon. When these two tendons have been divided, it is very important that the varus deformity should be thoroughly and over-corrected and immediately before any effort is made to cut the tendo-achilles, since the resistance offered by this large tendon better enables you to rotate and adduct the foot. This is done by forcible manipulation and massage. The tendo-achilles is then divided by subcutaneous tenotomy and the equinus deformity overcome, the more pronounced the retraction the better the results. The small open incisions seldom require suture, being dusted with iodoform and co-aptation maintained by a single strip of zinc oxide adhesive plaster. The dressings are light and simple, and care is then taken not to allow crowding of the dressing material between the ends of the severed tendons. A canton flannel roller bandage is then evenly applied from toes to knee, over which comes a plaster dressing. I prefer this flannel to cotton, since it can be more smoothly applied and less apt to roll up under the outside dressing. The toes should be watched after for any signs of undue pressure. If the correction of the deformity has been complete, or nearly so, this first dressing should be left on so long as it remains hard and perfect. At each subsequent dressing, the foot should be thoroughly manipulated and over-corrected, and the plaster reapplied. Usually, at the end of three or four months, the plaster is discarded, the deformity has been cured and a retension apparatus is then ordered to be worn for about one year as a safeguard against any return of the varus. The Taylor club-foot brace is the one usually employed by the writer. All of the cases upon which I have operated or directly assisted in have been in infants before walking period, and I believe the earlier the operation is performed the better the results and quicker the recovery. In that class of cases who can locomote, the best results are gotten by having them practice walking from the very first, since the body pressure tends to assist in curing the deformity and in forming new articular facets upon the implicated tarsal bone, and the necessity for a retension apparatus diminishes accordingly. There is no reaction from such an operation; no fever, swelling or pain, and as a result the dangers to pressure gangrene are slight. In some of the cases, difficulty was experienced in keeping the plaster case from slipping, in which instance the knee was slightly flexed and the dressing carried above this joint.

When you remember the pathological changes in the parts involved you will readily understand that the above operative procedures are purely preliminary and but a step towards the successful treatment and correction of the existing deformity. If we can overcome by brute force the resisting points as offered by the anticus and posticus muscles and the tendo-achilles, together with the astrogalo-scapoid and calcaneo-cuboid ligaments, how much more scientific, easier and less cruel is it to cut these tendons, for an anesthetic is required in every instance, and under modern aseptic methods the dangers from tenotomy are nil. Almost any surgeon can correct the deformity of club-foot, but unless the corrected position be maintained sufficiently long to insure changes and alterations in the facets of the tarsal bones, elongation of the contracted ligaments and a corresponding shortening of muscles and tendons on the outside of the foot, relapses will occur under any form of treatment. This corrected position is maintained under plaster paris dressing until all resistance becomes passive, and not until then should a retension apparatus, such as the Taylor brace, be applied and worn as a safeguard. In the marked equinus variety, the existing deformity can not be corrected except by tenotomy, and in the so-called bloodless operations paraded before the public the value of tenotomy is recognized and done and then silently covered up under a snugly fitting plaster dressing. In the hands of the experienced surgeon, the treatment of club-foot has been uniformly successful. The failures heretofore occurring after mynotomy and tenotomy have resulted from improper or want of proper after mechanical treatment, and such failures can not be used as an argument against the operation as a source of relief any more than can the operation for appendicitis in inexperienced hands, and which results in death to the patient, be used as a contra-indication against future surgical interference. The recent public demonstrations by the noted German clinician Lorenz have served only to demonstrate the absolute necessity of the employment of more force at the initial operation, and that the Phelps operation and others of still more extensive cutting are wholly unnecessary; but the operation by a manual force alone can never be an operation of choice, since it offers no single merit of claim over the well-established customs of modern aseptic surgery in the treatment of club-foot.

In the series of eight cases operated upon two were double equino varus, four single equino varus, three of which involved the right foot. All made a uniform recovery.

